# Hemophilic and Secondary Hemorrhages

**Published:** 1906-06-15

**Authors:** George Zederbaum

**Affiliations:** Charlotte, Mich.


### HEMOPHILIC AND SECONDARY HEMORRHAGES.

BY GEORGE ZEDERBAUM, D.D.S. CHARLOTTE, MICH.

Read before the Central Michigan Dental Society, February 22, 1906.

The object of this paper is to point out treatments that would be effective in checking hemorrhages, whether they be of the recurrent or the hemophilic variety, or perhaps both. Many times, and especially in emergency cases, we are unable to determine what kind of a hemorrhage we have to deal with, and we little care, though we should. We see a hemorrhage, and by all the laws of nature we know it must be checked at once, as the danger of hemorrhage is known to all; but to the infrequency of the real severe, dangerous variety of hemorrhages after tooth extracting, do I attribute their most dangerous effects. The less frequent any pathologic condition the more unfamiliar we are with it and the less our practical knowledge of successful ameliorative treatment.

I am prompted to write on this topic by the recent reports in this connection, and because of the apparent indifference with which the Dental Fraternity regards them, citing a few cases of my own.

Not over six months ago the newspapers, and afterward the Dental Journals confirmed the report from Three-Rivers, I believe that three boys of the same family died within a few years of each other.—Two from hemorrhages after tooth extraction, and one, from a mere scratch on the tongue. Another case, I recall, is that of Senator Mitchell of Oregon, of whom no doubt, most of you have read. The DENTAL REGISTER No. 1-'06., published the item mentioning the fact that Mr Mitchell was suffering from diabetes and that his recuperative abilities were decidedly low. Perhaps so, but the fact, however, still remains, four teeth were extracted and the man died from the direct result of uncheckable hemorrhage. I say ought we not to know more about conditions that bring about such disastrous results? Ought we to pass over such reports lightly?

I will take up but a few minutes of your time with the explanation of hemophilic phenomena or secondary hemorrhages, as there are many standard authors who go into detail far more than the time or space would permit here; but a few words will refresh your memory relative to these conditions.

Dr. Osler defines hemophilia as a hereditary constitutional fault, characterized by a tendency to uncontrollable bleeding from slight wounds. The fact that fatal hemorrhage might occur from trifling injuries, has been known for centuries. The hereditary transmission in this disease is a very odd feature, as it is transmitted invariably through the mother who is not herself a bleeder, but a daughter of one. This affection, therefore, is most commonly met with in males. Dr. Robert Cabbott, of Boston, says in the International Text Book of Surgery that this disease is at least twelve times more frequent in males than in females. The real cause of the disease is not known up to date; as, contrary to the opinion of many, the blood of a bleeder does not differ in consistency, constituency, nor coagulability from the blood of a healthy individual. It is admitted however, that the coats of the blood vessels of a bleeder are much thinner.

Secondary or recurrent hemorrhage can be diagnosed with a greater certainty inasmuch as it usually comes on a few hours after the primary hemorrhage has ceased, and comes on most frequently during the night following the day upon which the extraction was done. The bleeding may be from the apical artery, or may be a capillary oozing from the whole socket, much resembling the hemophilic variety. In some cases the hemorrhage is confined to the gum only, or now and then it may proceed from all three sources. The causes of secondary hemorrhage may be varied: It may be that the patient was of a hemophilic diathesis; the primary clot may have been unconsciously sucked away; or there may have been a fractured alveolus, or a sharp coronal portion of the tooth may have been left about the gingiva, and occasionally even a remaining root will cause a like hemorrhage.

From whatever cause, either the hemophilic or the secondary variety of hemorrhage may be sufficiently profuse to drain from a pint to a quart of blood within a few hours, causing great anxiety to the patient and his friends.

Now that we come to the treatment, I wish to cite the cases that came under my observation and treatment within the last year, and to which I have already made reference. For convenience I will mention two hemophilic cases first, and the two of secondary hemorrhage last:—

Case 1.—A boy of eleven, apparently anemic and poorly nourished had a deciduous upper molar removed without anesthetics. He was real brave during this, his first experience; the usual slight hemorrhage followed, and the patient departed within a few minutes. Four days later, he was brought in his mothers arms, being too weak to walk or even to stand up, with the following history: Everything appeared to be all right for a few hours. Then a slight oozing of blood was noticed (everything attempted by his parents to check it failed; and, as the boy gradually grew weaker and refused nourishment, his parents became alarmed and a physician was called at one A. M. the third night.) He succeeded in stopping it within ten minutes by applying cotton pads saturated with ferric chloride. Four hours afterward, however, the hemorrhage again returned and this time it was more profuse. Same physician was called again and gave treatment as before, ordering patient returned to the dentist who did the extracting, if the bleeding had not stopped permanently. Upon examination, I found and removed an ill smelling plug of cotton, and washed out opening with hot water. Then I saturated a convenient shaped tampon of cotton with adrenalin chloride (full strength) and, prior to placing it, distributed in the opening, by means of compressed air and a glass pipette, Monsell[s salt. Hemorrhage ceased and has not returned. Plug was carefully removed in 36 hours and the boy was placed on a tonic of iron and arsenic and recuperated nicely in due time. A thorough questioning revealed hemophilic tendency in boy ever since birth, and many of the antecedents were subject to the same condition.

Case 2.—Young man 21, very fair complexion; extremely thin, soft skin, had a second lower molar extracted one evening. Next noon he reported at office saying hemorrhage had not ceased. I examined socket and found all clear of clots and debris, and saw what I thought to be a profuse capillary oozing, welling up from all around the soft tissues. I used Monsell's salts and tamponed as before, extending the plug far beyond the level of the other teeth, to insure better retention and pressure. He came again at six that evening, when he was still bleeding. This time I removed the plug, curetted the whole socket and washed with boiling water and packed tightly as before. The hemorrhage subsided again, only to return in an hour. On the following morning he was still spitting blood as if the tooth had just been extracted. This time I restored to red heat cautery and used a copious quantity of Nitrate of Silver solution. Hemorrhage stopped and did not return. The young man had a hemophilic history, and in fact, one of experience had only to look at him, to suspect him as a possible bleeder.

Case 3.—A young married woman, chlorotic of the most confirmed type had an upper and a lower molar extraced. Acestoria was used with the usual excellent result. At the time of extraction there was no hemorrhage to speak of, presumably owing to shock. I had not seen nor heard of the patient again, until three days later, when at about ten o'clock in the evening, I was telephoned to come at once to check the hemorrhage ensuing from the upper emptied socket only. Upon arrival, I learned there had been a slight hemorrhage the first day after extraction: none the second day; but that, at six in the evening of the third day, hemorrhage appeared and try what they might, neither the parents nor the husband, a druggist, could check the flow for more than fifteen minutes at a time. Each time it returnedi t seemed more profuse. Examination revealed capillary oozing from the sockets of the two buccal roots. There were no spiculæ present. Having had almost a similar case just a few days previous, and same having turned out satisfactorily in but one treatment. I used same method as before modifying it but slightly owing to conditions being not as handy at the home of the patient as they were in my office. Instead of blowing in of Monsell's salt, I sprinkled the same over the tampon saturated with the Adrenalin solution. Hemorrhage ceased in about three minutes. I ordered two doses of Chloride of Iron of 20 drops each to be taken at intervals of two hours and departed. Imagine my astonishment when awakened by phone at 2:30 A. M. hemorrhage having again returned at 2:00. Found patient weak from loss of rest as well as from loss of blood and in a very excited condition. Used boiling water for ten minutes irrigating the sockets and re-packed as before. Hemorrhage ceased and I ordered tincture of Gelsemium 20 drops every hour for three hours, and also a quarter of a grain of codeine every hour for three doses. There was no recurrence of hemorrhage and the tampon was removed 2 days afterward. Patient took Echafolta Solution and a Pepto-Manganese of Iron solution for a month afterward and has recuperated nicely.

Case 4.—A man of 36, had five upper and three lower teeth removed with local anesthetic and hemorrhage ensued and in a few minutes was entirely checked. Next morning the patient reported at the office bleeding, but only from one socket. I placed a piece of cotton saturated in Perchloride of Iron as far as the depth of the socket would permit, and the hemorrahge ceased. Everything went well until he removed the tampon 48 hours later, when the hemorrhage returned. He called again and I stopped it as before. Next day I carefully removed the plug and there was no sign of blood, but three days afterward it came again, and this time I found a fractured piece of the alveolus, which readly came away and gave us no further trouble.

I cannot say why the physician in case number one failed to check permanently, nor why I failed at first with the other cases. Perhaps some other means would have been of better value, but I admit I did not know any better way. The *New York Medical Journal* reports a case of a hemophilic boy where all hemostatics, adrenalin included, failed to check a hemorrhage from a slight bruise on the foot. Finally the ovarian extract of sheep in two and a half grain doses, was given three times and the hemorrhage ceased. If this remedy proves successful I for one am not willing to be without it. In my limited experience, I find that a secondary hemorrhage is more easily controlled than one of the hemophilic variety. Many authors recommend internal use of perchloride of iron one dram every two hours and a purge of sulphate of sodium. Iron and Arsenic also can be freely given and kept up during the convalescence. Iron internally is a valuable adjunct to the external applications of all the mild hemostatics not injurious to the soft tissues.

I now make it an invariable rule when I am called to extract a tooth in the evening, to be cautious ahout the hemorrhage and plug the socket w th a hemostatic treatment for over night. My advice is to be cautious also when extracting for thin, soft skinned, pale faced boys, there is where you are apt to run amuck.

This point I wish to emphasize:—Each practitioner has his favorite manner in which he checks these hemorrhages but we will admit even the best of systems will occasionally fail; and, as the old saying goes, "What may cure John may sicken Paul." To know of just one way to do a thing and not in any other manner is but little better than not to know it at all; especially so in such serious cases as secondary hemorrhage after teeth extraction which according to Osler results fatally many times. I therefore would suggest that each professional brother who reads this article and who has had experience in checking secondary and hemophilic hemorrhages, would publish his particular manner of combating them, so that we may know of more than one or two good ways and *accessible* means of stopping such hemorrhages. One could try some other method if the first he used failed, and so on until a good result comes. If we are proud possessors of a light on any subject, let us not hide it under a bushel, but let us uncover it and pass it around.

